# An 8-mm port site hernia after robotic-assisted ileocecal resection: a case report

**DOI:** 10.1186/s40792-024-01878-x

**Published:** 2024-04-02

**Authors:** Changgi Ahn, Masatsune Shibutani, Kishu Kitayama, Hiroaki Kasashima, Yuichiro Miki, Mami Yoshii, Tatsunari Fukuoka, Tatsuro Tamura, Takahiro Toyokawa, Shigeru Lee, Kiyoshi Maeda

**Affiliations:** Department of Gastroenterological Surgery, Osaka Metropolitan University Graduate School of Medicine, 1-4-3 Asahi-machi Abeno-ku, Osaka City, Osaka Prefecture 545-8585 Japan

**Keywords:** Port site hernia, Robotic-assisted surgery, Ileocecal resection, Colon cancer

## Abstract

**Background:**

Robotic-assisted surgery is steadily becoming more prominent. The majority of reports regarding port site hernias (PSHs) have involved laparoscopic procedures. Currently, it is common to suture the fascia at port sites that are 10 mm or larger; however, the closure of 5-mm port sites is not considered mandatory. The da Vinci^®^ surgical system (Intuitive Surgical Inc., Sunnyvale, CA, USA) utilizes a distinctive 8-mm port. We report a case of an early-onset PSH at an 8-mm port site after robotic-assisted ileocecal resection.

**Case presentation:**

A 74-year-old male patient with a body mass index of 19.7 kg/m^2^ was diagnosed with cecal cancer and underwent robotic-assisted ileocecal resection. A 3-cm midline incision was made at the umbilicus for insufflation. Under laparoscopic visualization, three ports (12 mm, 8 mm, and 8 mm) were inserted in the lower abdomen. An 8-mm port was inserted in the left subcostal region, and a 5-mm port was inserted in the left lateral abdomen. The procedure was performed without significant intraoperative complications. The fascia was closed only at the umbilicus and 12-mm port site; the fascia at the 8-mm port sites was not closed. The patient was initially discharged without complications; however, on postoperative day 11, the patient was urgently hospitalized again because of PSH incarceration. After manual reduction, the fascia was sutured closed under local anesthesia. The hernial defect was small and barely allowed the insertion of a little finger. There was no evidence of compression or significant damage to the fascia. On postoperative day 27, the patient was discharged after experiencing good recovery.

**Conclusions:**

Robotic-assisted colectomy could contribute to the risk of PSHs because of its surgical characteristics. Although routine closure of the fascia at 8-mm port sites is not mandatory, it may be beneficial in certain cases.

## Background

Because robotic-assisted surgeries are fairly new, reports of their safety are scarce. Most existing reports of port site hernias (PSHs) have focused on laparoscopic procedures. However, the prevalence of robotic-assisted surgeries is increasing across various fields, thus necessitating the evaluation of the specific risks for PSHs associated with robotic procedures that are distinct from the risks associated with laparoscopic procedures.

Although closure of the fascia at port sites that are 10 mm or larger is recommended, closure of the fascia at 5-mm port sites is not considered mandatory [1]. The da Vinci^®^ surgical system (Intuitive Surgical Inc., Sunnyvale, CA, USA) uses an 8-mm port, which is an unprecedented size; therefore, the necessity to perform fascia closure at such port sites has been debated. Some studies have contended that there is no need to perform fascia closure at 8-mm port sites [2, 3]. However, because of the tendency for PSHs to cause intestinal strangulation, it is crucial to adequately evaluate them. We present a case of an early-onset PSH at the 8-mm port site after robotic-assisted ileocecal resection.

## Case presentation

A 74-year-old male patient presented with a sub-circumferential elevated lesion around the ileocecal valve. A detailed examination revealed findings that were strongly suggestive of cancer (biopsy grade group 4). The tumor progressed to the ileum, thereby making endoscopic treatment difficult and creating the risk of stenosis. Therefore, the patient was referred to our department for surgical intervention. The patient had a height of 163 cm, weight of 52.4 kg, body mass index of 19.7 kg/m^2^, and type 2 diabetes mellitus as a comorbidity. He had no history of surgery or smoking. Cecal cancer (cT1N0M0 cStage I) was diagnosed, and robotic-assisted ileocecal resection was performed.

**Fig. 1:**
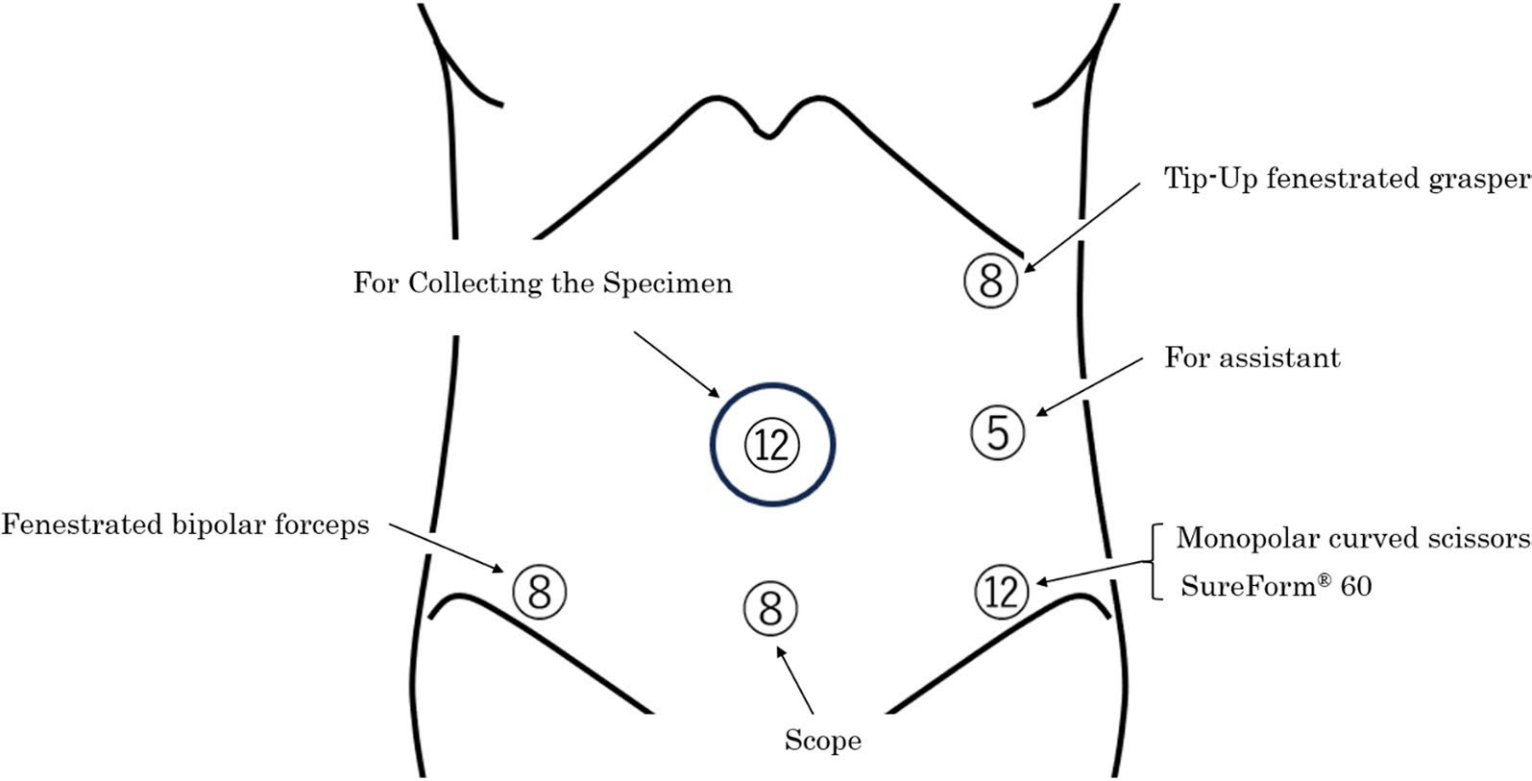
Port placement. The numbers in circles indicate the port sizes. A 3-cm incision at the umbilicus was made, but it remained unused during the console operation; it was specifically used to collect the specimen. One of the two 8-mm ports located in the lower abdomen is positioned in proximity to the iliac crest. The 8-mm port in the lower central abdomen serves as the scope port. The other ports, excluding the central 12-mm port and 5-mm port, are connected to the robotic arms

**Fig. 2:**
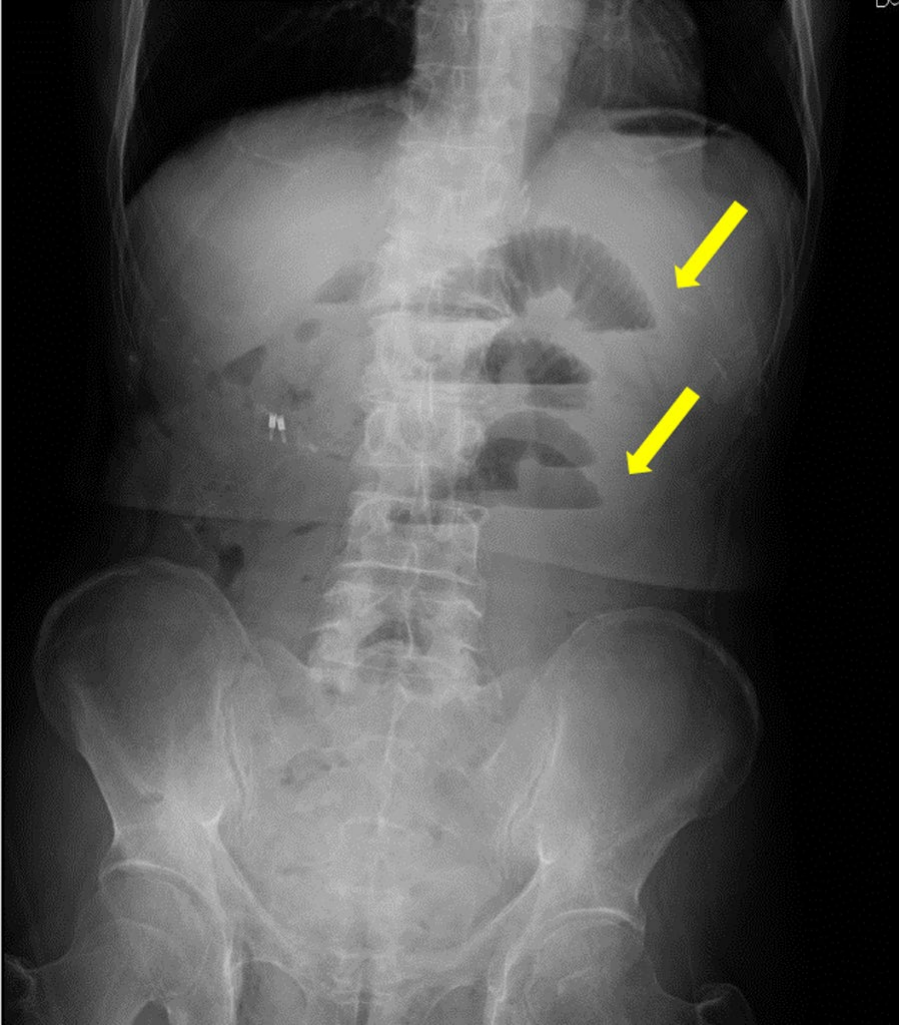
Abdominal radiography image. Upright anterior view. Dilated bowel loops with multiple air-fluid levels suggestive of intestinal obstruction are observed

**Fig. 3:**
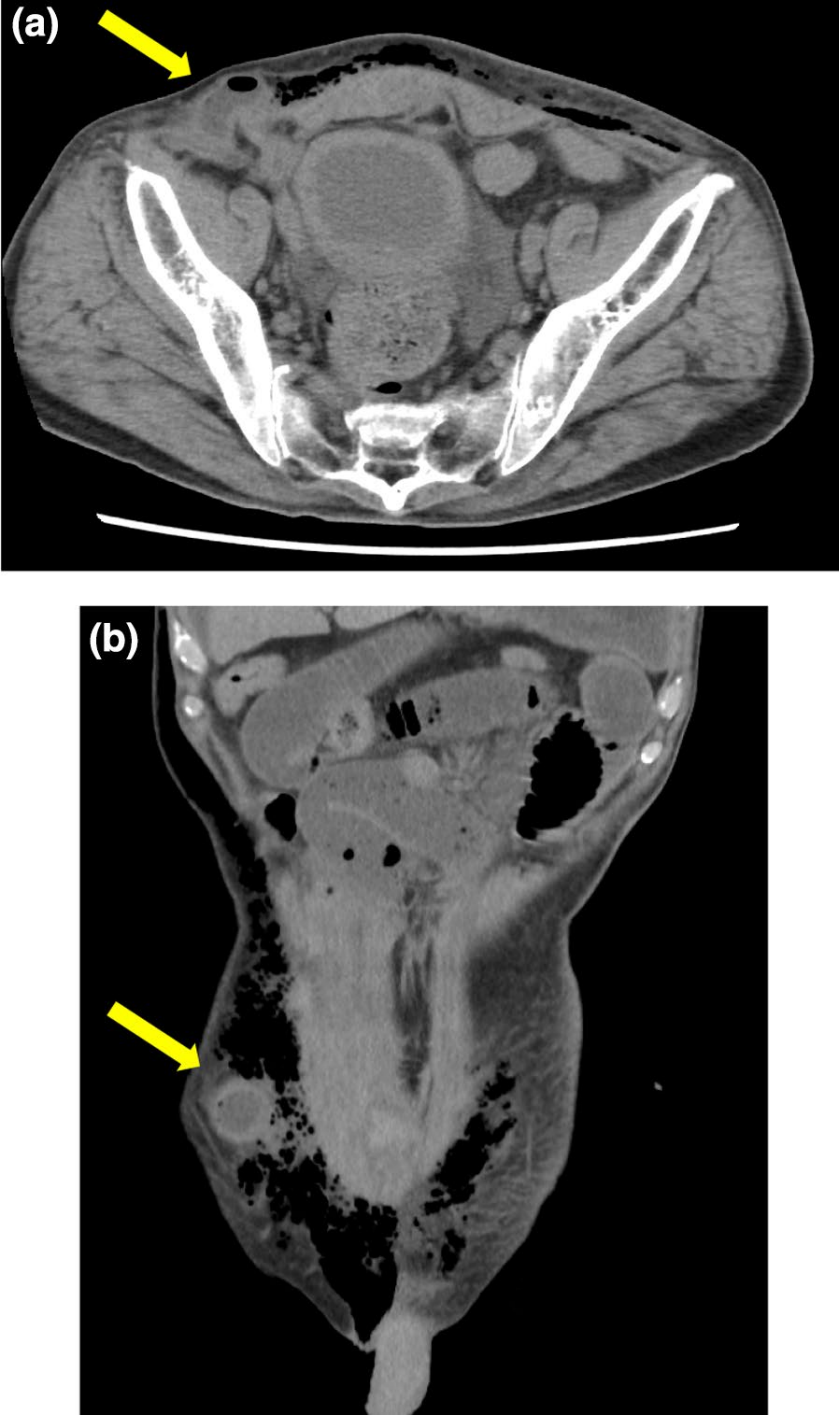
Plain abdominal computed tomography image. **a** Axial view. **b** Coronal view. The small intestine is protruding and incarcerated at the site of the 8-mm port in the lower right abdomen (yellow arrows)

The ports were arranged as shown in Fig. 1. Initially, a 3-cm midline incision was made at the umbilicus, and the TOP wound retractor (XS size; TOP Corporation, Tokyo, Japan) with Free Access (Xssize; TOP Corporation, Tokyo, Japan) and a 12-mm port were attached for insufflation. Under laparoscopic visualization, three ports (12 mm, 8 mm, and 8 mm) were inserted in the lower abdomen. Then, an 8-mm port was inserted in the left subcostal region, and a 5-mm port was inserted in the left lateral abdomen. The camera was inserted through the central 12-mm port, and the surgical field was developed using laparoscopic forceps. Subsequently, the da Vinci Xi^®^ patient cart (Intuitive Surgical Inc.) was manually rolled into position. The central 8-mm port in the lower abdomen served as the scope port, whereas the other two ports in the lower abdomen and the 8-mm port in the left subcostal region were connected to the robotic arms. The 12-mm port at the umbilicus remained unused during the console operation. Using a medial approach, the ileocecal artery and vein were tied off at the root. A colon-to-ileum anastomosis was performed through the 12-mm port using the overlap technique and SureForm^®^ 60 blue (Intuitive Surgical Inc.). The procedure was performed without significant intraoperative complications. No drainage tubes were used. The umbilical fascia was closed using a monofilament absorbable suture, and the fascia at the 12-mm port site was closed using a braided absorbable suture. At all 8-mm and 5-mm port sites, the fascia was not closed. The total surgical time was 127 min, and bleeding was minimal. There were no changes in the patient’s position during the console operation. The histopathological diagnosis was tubular adenocarcinoma (well-differentiated type) with an adenoma without cancer cells that did not infiltrate beyond the submucosal layer or lymph node metastasis. The pathological diagnosis revealed pTisN0M0 pStage 0. On postoperative day 3, the patient presented with bloody stools; therefore, a colonoscopy was performed. Although blood was observed on the anal side mucosa at the anastomotic site, ongoing bleeding was not identified, and special intervention for hemostasis was not required. The patient was discharged on postoperative day 9 without complications. However, on postoperative day 11, the patient presented to our emergency department with abdominal pain and vomiting. A physical examination revealed a thumb-sized bulging mass with tenderness at the right lower abdominal port site. Abdominal radiography revealed the findings suggestive of intestinal obstruction (Fig. 2), and plain abdominal computed tomography showed small bowel herniation and incarceration at the port site (Fig. 3a, b). After manual reduction, the patient was admitted to the hospital. Subsequently, the symptoms improved, but abdominal radiography showed limited improvement in small bowel dilatation. On postoperative day 16, the patient experienced vomiting again. Plain abdominal computed tomography showed small bowel herniation at the same location, suggesting incomplete reduction or repeat incarceration. Because of the relatively mild and gradual course of symptoms and ease of reduction under ultrasound visualization, it was determined that irreversible blood flow impairment was not present in the intestine. Under local anesthesia, a 2-cm skin incision was made, and the fascia was closed with a braided absorbable suture. Signs of hernial defect strictures and significant defects were not observed during closure. The small size of the hernial defect barely allowed the insertion of a little finger. Treatment was administered for aspiration pneumonia caused by vomiting. On postoperative day 27, the patient was discharged after experiencing good recovery.

## Discussion

According to Tonouchi et al., PSHs are classified into three types: the early-onset type, which often occurs within a few weeks after surgery and frequently leads to bowel obstruction; the late-onset type that develops several months after surgery; and the special type, indicating the protrusion of the intestine or omentum, or both [1]. In this case, the hernia developed 11 days postoperatively, presenting as the early-onset type with associated small bowel obstruction. During suturing under local anesthesia, the peritoneum was not identified as a hernia sac.

A PSH is a complication exclusive to laparoscopic surgery [4]. According to previous reports, its incidence ranges from 1.50% to 1.80% [5, 6]. Regarding the causes of PSHs, technical factors during port insertion, such as incisional length, the shape and angle of the trocar tip, unnecessary damage to the abdominal wall during port insertion, intraoperative weakening of the abdominal wall due to factors such as increased intraoperative pneumoperitoneal pressure and vigorous movement of the trocar, and decisions on whether to close the port site, all contribute. In addition, patient-related factors, such as a history of multiple pregnancies, advanced age, which represents a weakened abdominal wall, and medical conditions such as diabetes mellitus (a risk factor for delayed wound healing and postoperative infection), chronic respiratory diseases leading to chronically elevated intra-abdominal pressures, and obesity, have been reported [1, 7, 8]. Furthermore, new risk factors have emerged with the increasing prevalence of robotic-assisted surgeries, such as those involving the da Vinci^®^ surgical system (Intuitive Surgical Inc.). During laparoscopic surgery, ports are fixed to the abdominal wall, and the pivot point of their movement is inevitably the abdominal wall. However, during robotic-assisted surgery, ports are attached to robotic arms, and the pivot point for port movement is always the remote center. Therefore, unless the ports are connected to the robotic arms in a natural position, with no three-dimensional pressure on the abdominal wall, there will be a constant load on the abdominal wall during surgery, leading to fascia damage. Depressurizing after adjusting the remote center to the appropriate depth and attaching the ports to the robotic arms are recommended [9]. However, as many have suggested, the hypothesis that the port size is a significant risk factor for PSHs is reasonable [10–18]. During laparoscopic surgery, suturing the fascia at port sites that are 10 mm or larger is generally recommended; however, closure of the fascia at 5-mm ports is not routinely performed [1]. There is uncertainty regarding the need to close the fascia at 8-mm ports. Some reviews have suggested that closure of the fascia at such port sites may not be necessary; in actual clinical practice, many facilities do not perform closure of the fascia at 8-mm port sites [2, 3]. However, data regarding hernias associated with 8-mm port sites are insufficient.

Diez-Barroso et al. reported 178 robotic-assisted digestive surgeries performed at a single institution and found that the fascia was not closed at all 8-mm port sites; furthermore, only three (1.7%) of the 178 patients developed PSHs at the 8-mm port sites, accounting for 0.3% of all 8-mm ports (433 ports) [19]. In addition, Damani et al. analyzed 11,566 various robotic-assisted surgeries, including general surgery, urological surgery, and gynecological surgery, and found that the incidence of PSHs at 8-mm port sites was 0.1% (11 cases) [3]. These findings suggest a very low frequency of PSHs at 8-mm port sites. However, it is noteworthy that 10 of the 11 PSHs occurred specifically at the lateral abdominal port sites [3]. The target organ during robotic-assisted colectomy is located in the mid-abdomen, and ports are mainly placed in the lower abdomen. Therefore, the abdominal wall at the port sites is more susceptible to the effects of gravity and intra-abdominal pressure. In addition, during robotic surgery, maintaining a certain distance between ports is recommended to prevent collisions of the robotic arms [9]. Consequently, ports are often positioned more laterally beyond the Spigelian fascia. The internal and external oblique muscles, as well as the transversus abdominis, have origins and insertions from the ribs to the iliac crest and inguinal ligament. Because of the different directions of their muscle fibers, they move in different directions during insufflation and desufflation. The sliding phenomenon walls off the tract made by the port and prevents herniation after desufflation. However, muscle fibers closer to their origins or insertions may have limited mobility, making it less likely for this sliding mechanism to occur. In other words, in the lower abdomen, on the lateral side near the iliac crest, the risk of a PSH may be higher because the sliding phenomenon is less likely to occur [19]. Our patient did not have the above-mentioned patient-related risk factors. While we cannot deny the possibility of excessive damage to the abdominal wall during port insertion or unnecessary damage to the abdominal wall during the operation, this case had difficulties in the functioning of the “shutter mechanism,” not only from the perspective of port configuration but also due to the patient’s relatively slender body mass index of 19.5 kg/m^2^. This could have resulted in less mesenteric and preperitoneal fat, making it easier for the bowel to get entrapped in the fascial layer. Regarding reports on PSHs after robotic surgery, a review of individual cases did not reveal any specific considerations other than the tendency to occur in the lateral port sites. However, it is noteworthy that the majority of reported cases of PSHs occurred after urological and gynecological surgeries, with only one case reported after digestive surgery [7, 20–25]. This discrepancy is likely not disease-specific but rather attributed to the timing of the widespread adoption of robotic surgery. It is important to note that in the field of digestive surgery, where the adoption of robotic surgery has been slightly delayed compared to urology, there could be an increase in the number of reports on PSHs in the future.

Closure of the fascia at 12-mm port sites significantly reduces the incidence of PSHs; therefore, closure of the fascia at 8-mm port sites may be beneficial for preventing PSHs [26]. Thus, even though closing all the layers at 8-mm port sites is complex and not practical, closing the fascia alone may lead to a Richter-type hernia, so it is preferable to close all the layers of the abdominal wall. Moreover, the closure of an 8-mm port site under direct visualization is challenging; therefore, the use of specialized instruments may be advisable [27]. In addition, there are potential disadvantages associated with closure at 8-mm port sites, such as the risks of intestinal injury and damage to the abdominal wall vessels and nerves, including the inferior epigastric vessels [28]. Therefore, guidelines should be established to determine which cases with specific risk factors warrant closure of the fascia. However, statistical evaluations remain challenging because of the low incidence of PSHs.

## Conclusions

During robotic-assisted colectomy, the port is often placed in the lower lateral abdomen, creating the risk of a PSH. Because of the low incidence of PSHs, routine closure of the fascia at the 8-mm port site may not be necessary; however, it could be effective in certain cases. Further evaluation is necessary to determine the criteria for closure of the 8-mm port site.

## Data Availability

Not applicable.

## Abbreviation

PSH: Port site hernia
